# Indefinite Plasmonic Beam Engineering by In-plane Holography

**DOI:** 10.1038/srep28926

**Published:** 2016-06-30

**Authors:** J. Chen, L. Li, T. Li, S. N. Zhu

**Affiliations:** 1 National Laboratory of Solid State Microstructures, College of Engineering and Applied Sciences, School of Physics, Nanjing University, Nanjing 210093, China; 2 Collaborative Innovation Center of Advanced Microstructures, Nanjing University, Nanjing 210093, China

## Abstract

Recent advances in controlling the optical phase at the sub-wavelength scale by meta-structures offer unprecedented possibilities in the beam engineering, holograms, and even invisible cloaks. In despite of developments of plasmonic beam engineering for definite beams, here, we proposed a new holographic strategy by in-plane diffraction process to access indefinite plasmonic beams, where a counterintuitive oscillating beam was achieved at a free metal surface that is against the common recognition of light traveling. Beyond the conventional hologram, our approach emphasizes on the phase correlation on the target, and casts an in-depth insight into the beam formation as a kind of long depth-of-field object. Moreover, in contrast to previous plasmonic holography with space light as references, our approach is totally fulfilled in a planar dimension that offers a thoroughly compact manipulation of the plasmonic near-field and suggests new possibilities in nanophotonic designs.

Controlling the light propagation at will is what the people are always in pursuit of. In recent year, light beam has been discovered in novel forms with nondiffracting properties rather than the common Gaussian beam (e.g., Bessel beam^1^, Airy beam^2^,^3^, Mathieu and Webber beams^4^, etc.). Moreover, these novel beams have even been realized in the surface plasmon polaritons (SPP)-a bounded electromagnetic wave with strong field confinement at the metal surface, which enables people to manipulate the light at sub-wavelength scale in unconventional ways^5^,^6^,^7^,^8^,^9^. Among these progresses, the phase design was a key point, and the amplitude modulation was also considered more recently^10^, which are indeed consistent with the principle of optical holography.

Nowadays, optical holograms are undergoing a rapid development in three-dimensional (3D) imaging and colorful displays^11^,^12^,^13^,^14^ using plasmonic metasurfaces, owing to the artificial elements provide flexible pixel designs. In addition to these spatial holograms where the target and reference beams are both free space light, the near-field SPP wave has already been set as the reference beam^15^,^16^,^17^,^18^, or the target^9^,^10^,^19^,^20^, or even both^21^ in the recent progresses. Therefore, near-field hologram has stepped into a more popular view with powerful ability in near field routing and beam engineering. Although those impressive SPP beams have been demonstrated, their 2D holograms were usually encoded from a mathematically derived phase^20^ (and amplitudes^9^,^10^) that belong to definite solutions of wave equations or trajectory functions. However, from another point of view, can a propagating beam be regarded as a collection of discrete point objects (see the scheme in Fig. 1a)? If yes, one would suppose to use a group of point holograms to build any type of beam (even any field distribution) that would go far beyond the definite forms.

In this paper, we intensively analyzed the holographic beam engineering in an in-plane plasmonic scheme, and found the critical importance of the phase correlation of a longitudinal target (e.g., a propagating beam) in a holographic process. As an example, we proposed and realized an indefinite plasmonic beam that propagates in a trigonometric function of sine-oscillation, which is absolutely against any solution of the free space beams. By carefully investigation, it is concluded that the amplitude distribution of the source (corresponding to conventional phase mask) plays an important role in the formation of high quality oscillating beam, which was usually ignored in conventional holograms and caustic beam designs. Our research deepens the understanding of plasmonic beam formation in a holographic perspective, and would enrich people more possibilities in handling the optical field in holographic display, optical trapping, etc.

## Results

### Holographic design of an oscillating beam

**Figure 1:**
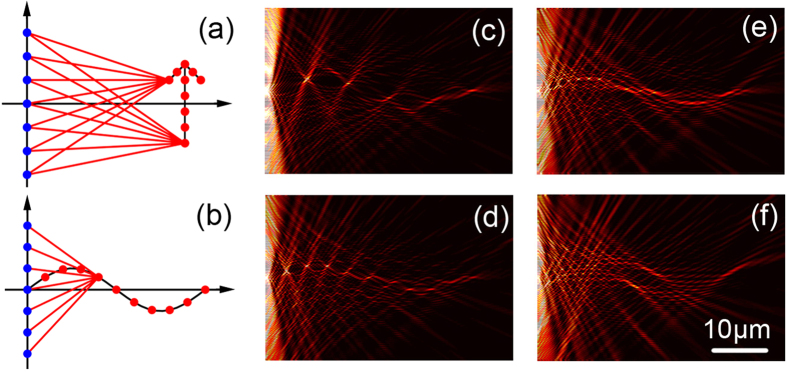
Schematic of source derivation in two-dimensional holography and theoretical calculation of independent points reconstruction. (**a**) Schematic illustration of source derivation from the object sample points and (**b**) source detivation of an oscillating beam. The reconstruction result of N independent points along beam’s trajectory, (**c**) N = 5, (**d**) N = 10, (**e**) N = 20, (**f**) N = 25.

Since considerable self-bending beams have been designed and revealed in free space and plasmonic regimes^2^,^3^,^4^,^5^,^6^,^7^,^8^,^9^,^10^, a straightforward challenge is whether a beam can be twisted to be an oscillation form. It also appears to challenge the people’s common recognition of the light traveling in free space. To make it clear, therefore, a sine function trajectory for a propagating SPP beam is preliminary proposed with the oscillating amplitude of 3 μm and period of 50 μm (
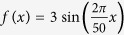
). This beam can be considered as composition of *N* discrete target points within a propagation distance of an oscillation period (50 μm). Here, we define the oscillating beam propagates along *x* direction, which is contributed from the sources arranged in *y* axis with particular phase and amplitude modulation (see Fig. 1b). According to the reversal process, the holographic SPP field of the sources in *x*-axis can be calculated by summarizing all the radiations from those local virtual points as^22^


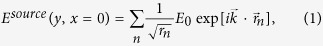


where *r*_*n*_ is the distance between the *n*th point in beam and the real source position in *y*-axis. Firstly, we define these virtue points are independent without particular phase correlation for a propagating wave. So that, a fixed phase of φ_0_ is set for all target points in retrieving the source profile. In this case, a set of diffraction processes with respect to N = 5, 10, 20, and 25, are theoretically obtained by numerical calculations (see Fig. 1c–f). It can be seen that in the sparse designs (N = 5, 10, 20) the desired target points are clearly focused ready to form a beam, while in the denser one (N = 25) the beam formation collapses. This interesting phenomenon would account for the improper initial phase setting of these target points. From the results of Fig. 1c–e, we may find these target points are well independent without connecting field, indicating they are unable to form a continuous beam. So the collapse in a denser point case is the destination, where a natural phase evolution is not provided as the two virtue points of the beam are close enough.

**Figure 2:**
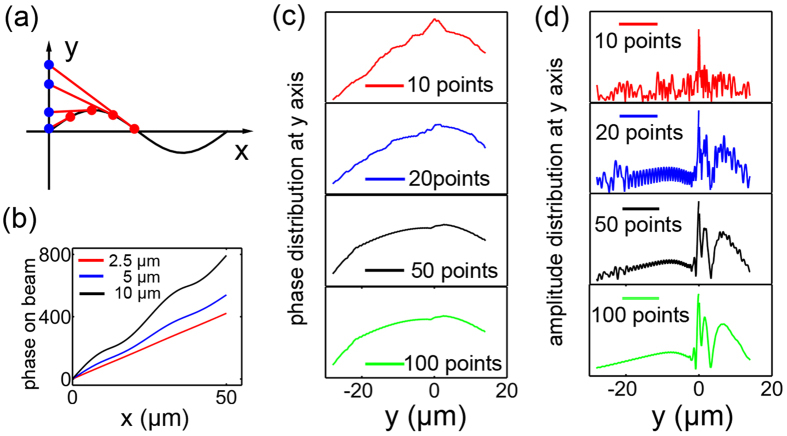
Schematic of caustic beam design and parameters retrieved from the holography method. (**a**) Schematic of caustic curve that constructed by the geomerical rays tangent to the curve. (**b**) Phase evolution in *x* along the beam propagation with different oscillation amplitudes (2.5 μm, 10 μm and 20 μm). (**c**) Phase and (**d**) amplitude distribution in y-axis derived by our holography method with different number of virtual points selected along beam’s trajectory.

With this concept kept in mind, we need to work out a precise phase evolution along such an indefinite oscillating beam. Although the sine-function trajectory does not satisfy any solution of wave equation, fortunately, the geometric caustic beam design provides us a convenient method to deduce the phase evolution^23^,^24^. The derivation is based on the principle that the beam in a caustic curve can be constructed by multiple geometrical rays that tangent to the curve itself, shown in Fig. 2a. For example of the sine-oscillation beam trajectory of *f*(*x*) = sin(*x*), we can determine the spatial phase distribution *ψ*(*y*) in the start line *x* = 0 by integrating the phase equation as^23^


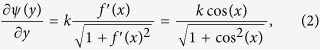


where *k* is the value of wave vector. It is easy to deduce the phase evolution of the target beam along its propagation as


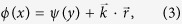


where *r* is the optical path along the tangent line with respect to every local point in the beam and correspondent source.

Figure 2b shows nonlinear relations between *ϕ*(*x*) and *x* of beams with different oscillating amplitudes, which indicate that the phase evolution along the *x* direction depends on the oscillation of the beam. After we get the phase on the beam *ϕ*(*x*), equation (1) used to calculate the distribution of amplitudes (*A*) and phases (*ψ*) of the real sources arranged along *y*-axis, will be rewritten as


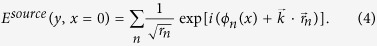


**Figure 3:**
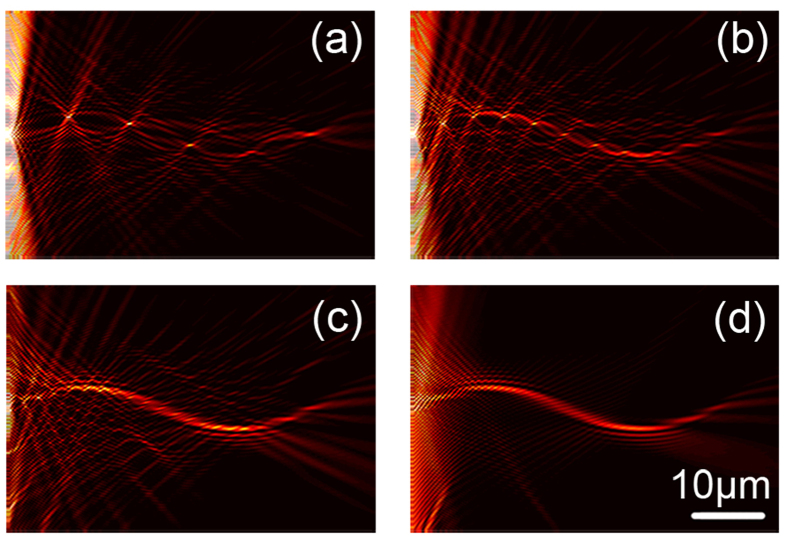
Reconstruction result by our holography method. Theoretical calculation of N phase collerated points along beam’s trajectory, (**a**) N = 5, (**b**) N = 10, (**c**) N = 20, (**d**) N = 50.

With equation (4), the information of sources along y-axis (*E*^*source*^) can be retrieved from N virtual points of target beam, where the phase and amplitude distributions are convergent when the number of points is large enough, as shown in Fig. 2c,d. It indicates the virtue points group with large enough density (N > 50) of is able to be considered as a continuous beam, which is consistent with the physical fact. Therefore, we calculated the whole diffraction processes with respect to N = 5, 10, 20, and 50, as the results shown in Fig. 3a–d. It is evident that the calculated sine-oscillating beam becomes better and better as the setting points increases, which rightly validates our holographic design with respect to a group of phase-correlated target points. This design is flexible and not limited to such a sine-oscillation trajectory. For examples, further amplitude-changed oscillating beams are also designed and revealed by calculations (see Supplementary Information), confirming the generality of our strategy.

### Experimental results

Based on the holographic design, the required phase and amplitude distributions of a sine-oscillating beam have been obtained, and the next step is to fulfill it in experiment. The non-perfectly-matched Bragg diffraction provides us a convenient method to manipulate those well defined SPP beams by a totally in-plane process^7^,^22^,^25^,^26^. However, only the phase distributions were mainly controlled in previous works. In this holographic process, the amplitude distribution of the diffraction process would as same important as the phase does. So, this in-plane process should be developed to load the amplitude information.

According to the in-plane diffraction method^7^, a well-defined nanohole array with a period of *a*_*x*_ = 610 nm in *x* direction and a various lattice (*a*_*y*_) in *y* direction are designed, where the parameter *a*_*y*_ is retrieve by solving equation


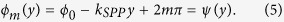


**Figure 4:**
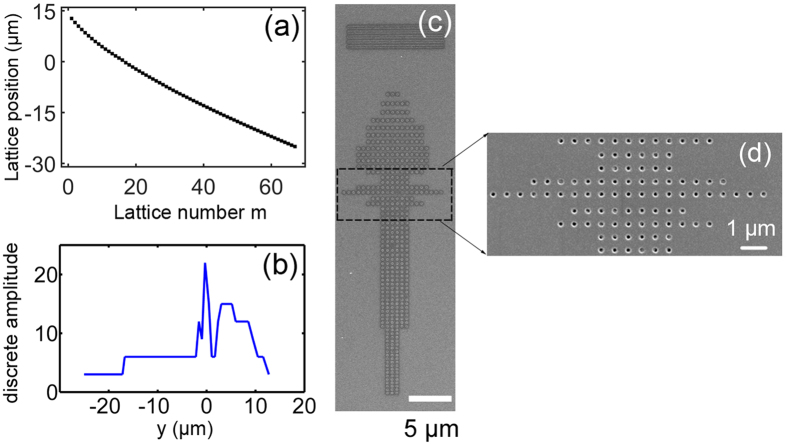
Micro/nano structure design and fabrication. (**a**) Lattice locations in y direction designed by in-plane diffraction process according the required phase distribution. (**b**) Discrete amplitude distribution by our holography strategy, which corresponds to the number of diffraction units designed in experiment. (**c**) SEM image of the sample fabricated by the focused ion beam with a controlled hole amount in every row. (**d**) Zoom-in image of partial of the sample.

Here *ϕ*_*m*_ is the discrete phases at the *m*th local lattice and *ψ*(*y*) is the required lateral phase distribution designed by the holography (see Fig. 2c). According to an oscillating beam with a trajectory of 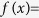

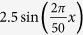
, the parameter of *a*_*y*_ is achieved by equation (4), and the corresponding locations of lattices are shown in Fig. 4a. In a common occasion, this nanohole array is designed with a fixed hole number in each row with respect to a homogenous diffraction intensity approximately. However, in order to control the local intensity for diffractions, a variation is introduced in every row, where the diffraction intensity is supposed to be proportion to the number of diffraction units. Since the diffraction process by the nanohole array should be discrete ones, the continuous amplitude distribution is therefore discretized firstly, as shown in Fig. 4b, which is ready to be imported into the sample by controlling the number of nanoholes in every row. Figure 4c shows the scanning electric microscope (SEM) image of the controlled sample designed for the sine-oscillating beam, and the details can be clearly observed in the zoom-in image Fig. 4d.

**Figure 5:**
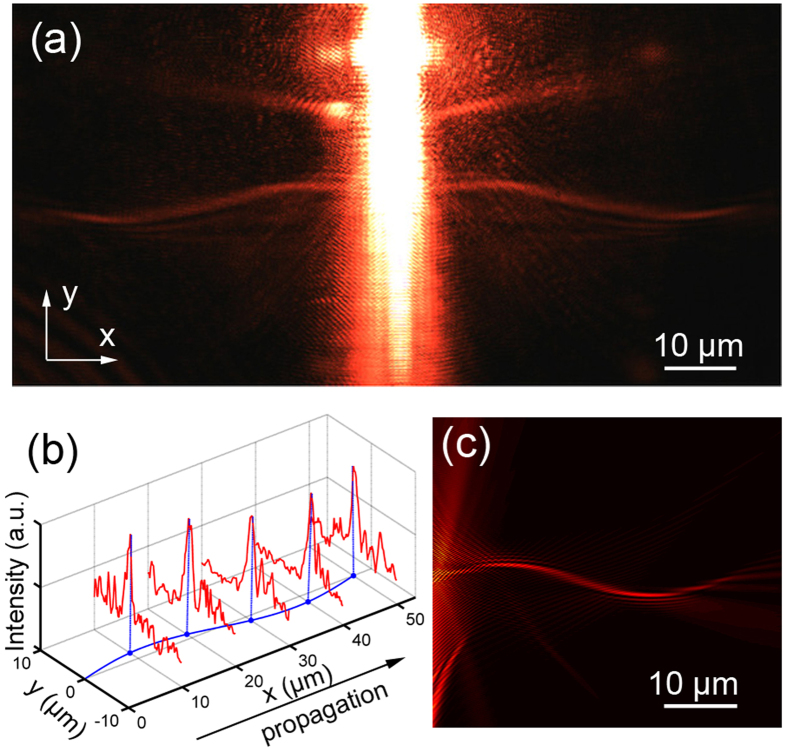
Optical characterization of the sine-ossilliating SPP beams. (**a**) The overall result of the sine-oscillating beam formation by diffractions recorded by the leakage radiation microscope system with an oil-immersed objective (NA = 1.42). (**b**) Extracted lateral field intensity profiles along the beam propragating, showing preserved beam peak (~1.3 μm) and osscilating trajectory (amplitude ~2.48 μm). (**c**) The corresponding theoretical result for comparision.

Figure 5a shows the experiment result observed by a LRM system^27^,^28^, where two branches of curved oscillating SPP beams from the center nanohole array are clearly manifested. For a clearer characterization of the achieved SPP beam, the cross-section profiles at different propagation distances of the right branch beam are plotted in Fig. 5b, where a remarkable intensity peak is manifested with preserved narrow beam width (~1.3 μm) and sine oscillation (amplitude about ~2.48 μm). A theoretical calculation result is shown in Fig. 5c for a comparison, where a very good coincidence is obtained indicating the successful realization of such an indefinite beam by the in-plane holographic approach. This good beam quality in micro-scale with intensive narrow peak and considerable small noises indicates potential applications in further near field routine and opto-mechanics designs.

## Discussions

Descritizing a propagating beam into multiple points has been successfully demonstrated in 3D holography by metasurface^12^, where the phase correlations between the points were not clearly clarified. In this work, it has been well manifested that an approximate beam shape will still be formed with a limited point density even lacking particular phase design, as shown in Fig. 1c–f. However, this design would inevitably introduce stray light since the fields from other points are rather dispersive with respect to a certain focal plane being checked. The lack of necessary phase evolution between those target points restricts more precise level of the holographic image. As for the caustic beam design by geometric optics, it has already gained great successes in self-bending beams recently^20^, however, the ignorance of the amplitude information makes it powerless in achieving those indefinite beams. A detailed comparison between the results of sine-oscillation beam by our holography and caustic beam designs are provided in the Supplementary Online Information both in calculations and experiments, which clearly shows the insufficiency of the caustic design.

In summary, we have successfully developed an amplitude hologram to realize an indefinite plasmonic oscillating beam, which is totally implemented in an in-plane process. The underlying mechanism of the beam formation has been emphatically investigated from the viewpoint of discrete virtue points for holography, where the phase correlation is discovered, for the first time to our knowledge, playing a critical role. In experiments, in-plane diffraction method was further upgraded to an intensity controlled process in diffractions to build up the high quality oscillating beam. In principle, this new strategy is not limited in achieving such kind of oscillating SPP beam, but wider indefinite beam engineering or optical controlling depending on one’s imaginations. Finally, comparisons between our strategy and 3D holography and caustic beam designs were addressed and discussed. Our study offers a unique insight into the plasmonic holography for beam engineering and is expected to inspire more intriguing phenomena and potential applications in beam engineering and optical manipulations.

## Methods

In experiment, the required phase and amplitude distributions of an oscillating beam are stored in a well-defined nanohole array. All the holes are about 200 nm in diameter and 20 nm in depth, which were fabricated by focus ion beam (FIB dual-beam FEI Helios 600 i) on an 80 nm thick silver film with a silica substrate. A grating with period of 610 nm besides the array is introduced to couple the incident laser to in-plane propagating SPP. When the SPP wave propagates through the hole array, it will be both diffracted out as radiation light and still confined on the metal surface as diffracted SPP waves. The property of diffracted SPP waves will be determined by the hole array, which can be characterized by Leakage radiation microscopy (LRM)^27^,^28^. The LRM is based on the detection of coherent leaking of SPP waves through the substrate, when the metal film is thin enough (usually below 100 nm) and the substrate optical constant is higher than that of the superstrate medium, which has been widely used in analyzing SPP propagations on thin metal films^7^,^22^,^25^,^26^ and nanowires^29^,^30^. In our LRM optical characterization, a He-Ne laser (λ0 = 632.8 nm) was coupled to the silver surface by the grating to form a plane SPP wave, which then propagates into the nanohole array and be diffracted. The overall result of SPP diffraction and the beam formation is recorded by an oil-immersed objective (NA = 1.42).

## Additional Information

**How to cite this article**: Chen, J. *et al*. Indefinite Plasmonic Beam Engineering by In-plane Holography. *Sci. Rep.* **6**, 28926; doi: 10.1038/srep28926 (2016).

## Supplementary Material

Supplementary Information
