# Supplementary material for: Indefinite Plasmonic Beam Engineering by In-plane Holography
Source: Sci Rep. 2016 Jun 30;6:28926. doi: 10.1038/srep28926 (PMC4928043; doi:10.1038/srep28926)
Supplement: Supplementary Information [file srep28926-s1.doc]

**Indefinite Plasmonic Beam Engineering by In-plane Holography**

J. Chen, L. Li, T. Li,* and S. N. Zhu

National Laboratory of Solid State Microstructures, College of Engineering and Applied Sciences, School of Physics, N­­anjing University, Nanjing 210093, China

*Collaborative Innovation Center of Advanced Microstructures, Nanjing University, Nanjing 210093, China*

**Supplementary information**

1. **Realization of beams with complicated shapes**

Here, we design and reveal some beams with more complicated shapes than in calculation. We first design an oscillation beam with different amplitudes at each section as

$f(x)=\left\{\begin{matrix} 4\sin(\frac{\pi}{15}x), \mathop{}\limits x<=15 \\ \text{ -8sin[}\frac{\pi}{30}\text{(}x\text{-15)],}\mathop{}\limits\text{15<}x\text{<=45 } \end{matrix}\right.$ . (S1)

It can be easily seen that the beam oscillates with amplitude of 4 μm and semi-period of 15 μm at the first section, while with amplitude of 8 μm and semi-period of 30 μm at the second section. We also design a beam oscillates with bigger amplitude at the first section than the second one as

$f(x)=\left\{\begin{matrix} 8\sin(\frac{\pi}{30}x), \mathop{}\limits x<=30 \\ \text{ -4sin[}\frac{\pi}{15}\text{(}x\text{-30)],}\mathop{}\limits\text{30<}x\text{<=45 } \end{matrix}\right.$. (S2)

The simulation results of these two beams are shown in Fig. S1a and Fig. S1b.

In order to confirm the generality of our strategy, more complicated beams with three sections along their trajectories are also designed. Figure S1c shows the simulation results of beams with trajectories as

$f(x)=\left\{\begin{matrix} 3\sin(\frac{\pi}{15}x), \mathop{}\limits\mathop{ }\limits x<=15 \\ \begin{matrix} \text{ -5sin[}\frac{\pi}{25}\text{(}x\text{-15)], 15<}x\text{<=40} \\ \text{ 10sin[}\frac{\pi}{50}\text{(}x\text{-40)], 40<}x\text{<=90 } \end{matrix} \end{matrix}\right.$ . (S3)

From these figures, we can find that beams really oscillates as what we design.


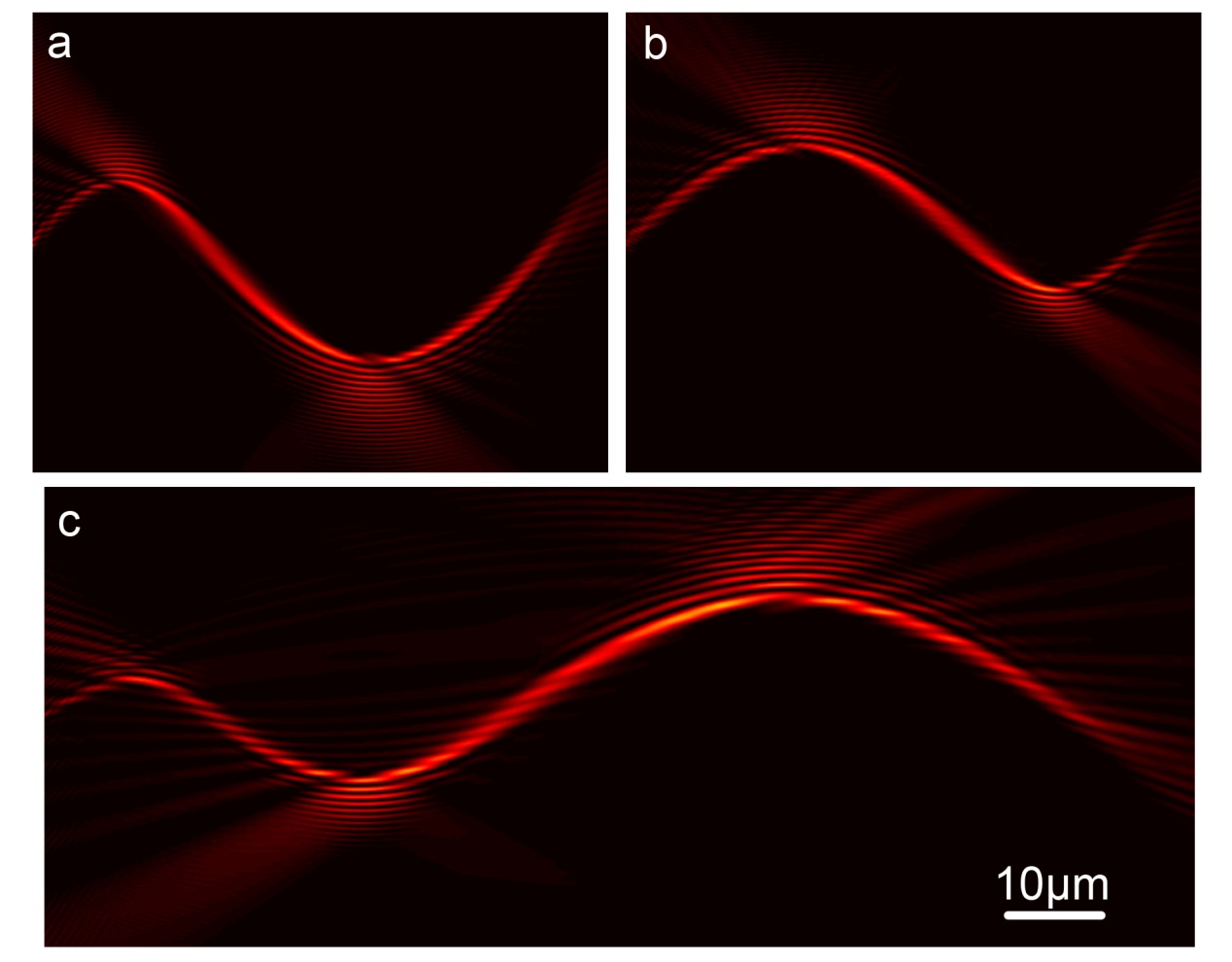


**Figure S1. Calculation of complicated oscillating beams by our holography method.** Construction result of beams defined as **(a)** equation (S1), **(b)** equation (S2) and **(c)** equation (S3).

**2. Comparison between caustic method and our strategy**

In caustic method the phase distribution of a desired beam can be derived by equation (2). According to the derived sources, we theoretically calculated the Sine-oscillating beam with a trajectory of $f(x)=3\sin(\frac{2\pi}{50}x)$ by Huygens-Fresnel integration. As shown in Fig. S2a, the oscillating shape can be seen. However, compared with the beam derived by our holography method [as shown in Fig. S2b], this oscillating beam shows many parasitic wave noises, especially in the vicinity of two curve inflections. In fact, this poor beam quality accounts for the design principle of the caustic beam. Because in that design, the local beam is closely related with the local sources in its tangent direction, so that the local beam around the inflections should have stronger intensities due to the similar tangent value, and result in intensive parasitic traces along these directions.

In experiment, we also use the non-perfect Bragg diffraction process to realize a beam with a trajectory of $f(x)=3\sin(\frac{2\pi}{50}x)$, derived by the caustic method. As in caustic method, we only need to care about the phase distribution, the sample corresponding to caustic method is a uniform array design, as shown in Fig. S2c. The right branch of the experiment result is displayed in Fig. S2d. To compare the experiment results, Fig. S2e is the right branch of beam shown in Fig. 4(d), which is derived by our strategy.

Here, we use a normalized mean square error (NMSE) to evaluate the beam quality, which is defined by

$NMSE=\frac{\sum\sum\left|I'(y,x)-I(y,x)\right|^{2}}{\sum\sum\left|I(y,x)\right|^{2}}$ , (S4)

where *I'*(*y*,*x*) is the intensity of all pixels in calculated propagation map and *I*(*y*,*x*) is the only intensity of pixels that rightly locate within the curved beam trajectory. From the equation, it is found that the value of NMSE should range from 0 to ∞, and the smaller NMSE value, the better beam quality (NMSE=0 means resultant beam completely matches the design). To further compare the beam qualities designed by caustic (Fig. S2d) and by holography (Fig. S2e), in its propagations more clearly, we modify the NMSE as a function of propagation distance (x):

$NMSE(x)=\frac{\sum\left|I'(y)-I(y)\right|^{2}}{\sum\left|I(y)\right|^{2}}$ , (S5)

as the results shown in Fig. S2f. It is evident that holographic design produced a much better Sine oscillating beam with considerable low NMSE values, especially in the region around the inflexions.


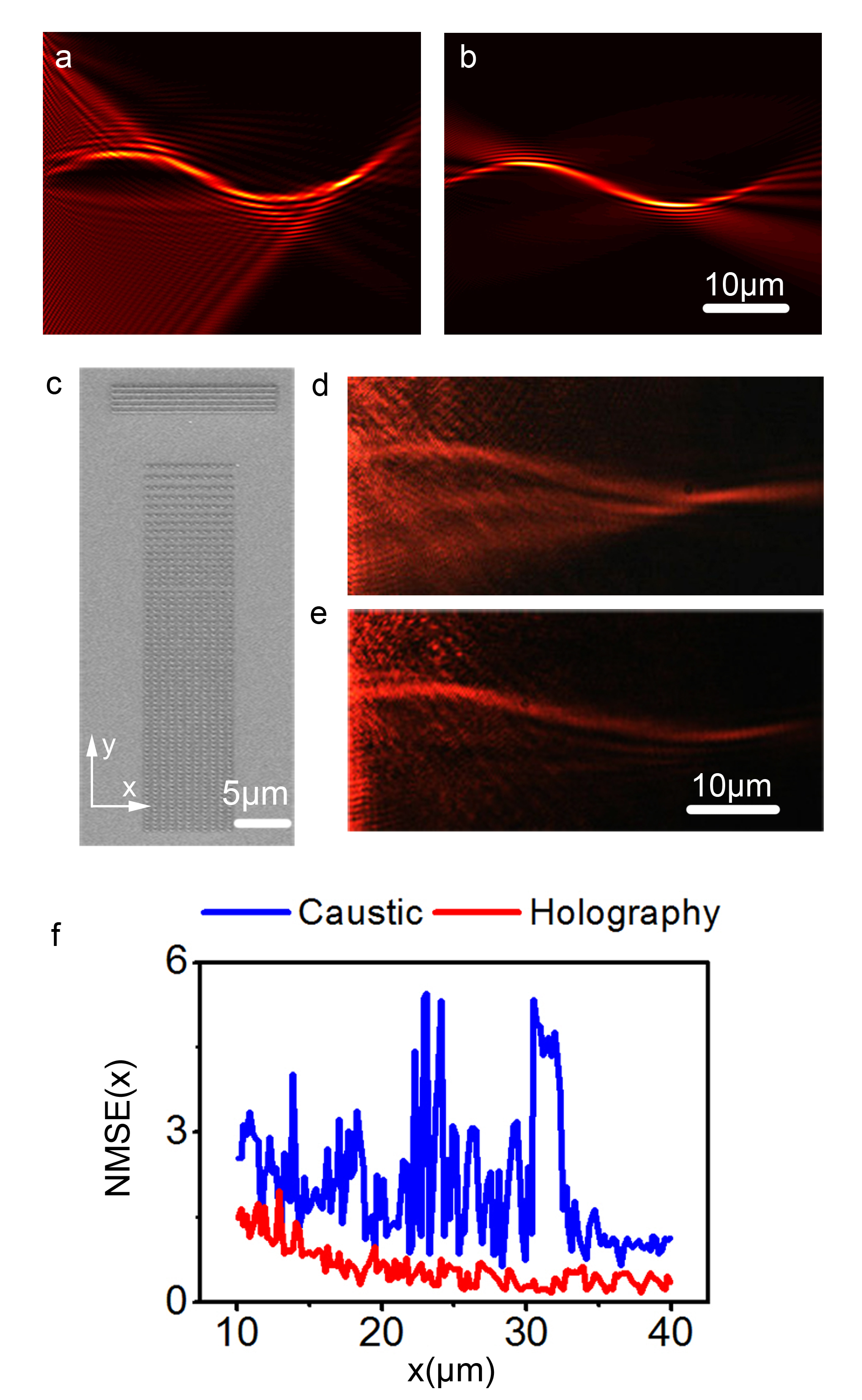


**Figure S2. Comparison of beams by caustic design and holography design.** Calculation of the Sine-oscillating beam by **(a)** caustic design and **(b)** holography design. **(c)** The SEM image of caustic design sample. The right branch of SPP Sine-oscillating beams derived by **(d)** caustic method and **(e)** holography method. **(f)** The NMSE (defined as equation (S5)) values derived from caustic method (the blue curve) and indefinite holography method (the red curve), as the function of propagation distance z.
